# Washed microbiota transplantation: a case report of clinical success with skin and gut microbiota improvement in an adolescent boy with atopic dermatitis

**DOI:** 10.3389/fimmu.2023.1275427

**Published:** 2023-11-15

**Authors:** Wan-Ying Deng, Wen-Jia Chen, Hao-Jie Zhong, Li-Hao Wu, Xing-Xiang He

**Affiliations:** ^1^ Department of Dermatology, The First Affiliated Hospital of Guangdong Pharmaceutical University, Guangzhou, China; ^2^ Department of Gastroenterology, Research Center for Engineering Techniques of Microbiota-Targeted Therapies of Guangdong Province, The First Affiliated Hospital of Guangdong Pharmaceutical University, Guangzhou, China; ^3^ Department of Hepatobiliary and Pancreatic Surgery, The First Affiliated Hospital of Shenzhen University, Shenzhen Second People’s Hospital, Shenzhen, China

**Keywords:** atopic dermatitis, washed microbiota transplantation, microbiome, intestinal microbiota, skin, case report

## Abstract

Atopic dermatitis (AD) is a chronic, recurrent inflammatory disease characterized by itching. The gut microbiome can help maintain skin immune homeostasis by regulating innate and adaptive immunity. Here, we report a case of AD in a 15-year-old adolescent boy who benefited from washed microbiota transplantation (WMT). WMT was performed for three courses, with each course lasting for three consecutive days and an interval of one month between two courses. Clinical assessments were conducted at each WMT course, and skin, blood, and stool samples were collected for microbial analysis. After three months of WMT treatment, the boy’s itchiness was effectively controlled: his skin showed noticeable improvement, with reduced *Staphylococcus aureus* in the skin lesions. The scores of SCORAD (SCORing Atopic Dermatitis), EASI (Eczema Area and Severity Index), NRS (Numerical Rating Scale), and DLQI (Dermatology Life Quality Index) significantly decreased compared to the baseline. Serum levels of eosinophil ratio, tumor necrotic factor-α, and interleukin-6 also reduced to the normal levels. There was a significant decrease in *S. aureus* in the skin lesions. Additionally, the intestinal flora became more diverse, and the abundance of *Bifidobacterium* species, significantly increased after WMT. No adverse events were reported during the treatment and the 1-year follow-up period. This case report provides direct clinical evidence for WMT as a novel promising treatment strategy for AD, and preliminary experimental data suggests the existence of an intestinal-skin axis in terms of the gut microbiota and the skin immune homeostasis.

## Introduction

Atopic dermatitis (AD) is a chronic, recurrent inflammatory disease with persistent itching, affecting approximately 15–20% of children and 1–3% of adults worldwide (1). The development of AD involves various factors, including genetic predisposition, impaired skin barrier function, altered immune response, and disrupted skin microbial balance (2, 3). The standard treatment for AD is the administration of topical corticosteroids, which are, however, often accompanied by adverse effects such as growth suppression in children, osteoporosis, telangiectasia, and skin thinning (4, 5). Recently, studies have found that gut microbiota composition of AD patients is significantly different from that of healthy controls (6), and the gut microbiota plays an important role in maintaining skin immune homeostasis by regulating innate and adaptive immunity (7, 8). Therefore, reshaping the gut microbiota, such as through fecal microbiota transplantation (FMT), may be a promising therapeutic option for AD. Indeed, Mashiah et al. reported the therapeutic effects of FMT in nine adult patients with moderate to severe AD, with a response rate of 77% (9). Here we present a case of a 15-year-old adolescent boy who benefited from washed microbiota transplantation (WMT), a modified form of FMT.

## Case report

In June 2022, a 15-year-old boy was admitted to our department due to a 9-year history of rash on his trunk and extremities, accompanied by intense itching. His mother had a history of urticaria, and his grandmother had a history of eczema. The patient typically had bowel movements 2-3 times weekly with difficulty in defecation. Physical examination revealed several scattered lesions on the body, characterized by redness, scabs, scaling, and local skin thickening. The patient was allergic to milk, freshwater fish, sesame seeds, animal hair, and pollen. Before the visit, he had been taking various antihistamines (such as cetirizine and ebastine), topical glucocorticosteroid creams (such as mometasone furoate cream), and calcineurin inhibitors (such as tacrolimus ointment), which had occasionally improved the rash; however, the symptoms continued to recur within days or weeks. His parents sought alternative medicines and visited our department specifically hoping to try WMT, which was well-performed in our department. Based on medical history, clinical manifestations, and physical examinations, AD was diagnosed for this patient according to Williams’ criteria and was graded as severe according to the SCORing Atopic Dermatitis (SCORAD) index. The patient received WMT treatment without the use of any topical interventions.

WMT procedure was performed as previously described (10, 11). Briefly, a group of healthy adults aged 25-30 years who have no bad habits (*e.g.*, smoking, alcohol addiction, *etc.*), no medications within the last 3 months, no history of diseases affectign gut microbiota were potential healthy donors. They were screened with a questionnaire and stool and blood tests were performed to rule out skin diseases, infectious diseases or any underlying health conditions. A fresh fecal sample was collected from a qualified donor using a sterilized container and purified by an automatic purification system based on GenFMTer (FMT Medical, Nanjing, China). After multiple rounds of centrifugation and sedimentation, the microbial pellet was resuspended in saline to obtain fresh suspension. Before each WMT course, a trans-endoscopic enteral tube (TET) was placed in the lower gastrointestinal tract through an endoscopic procedure and the fresh suspension was then delivered through the TET (12). WMT was performed for three courses, with each course containing one WMT procedure per day for three consecutive days and an interval of one month between two courses. The TET was removed after each course.

After each course, the patient’s skin was examined using a dermatoscope IDS-1100 (Illuco Corporation Ltd., Gyeonggi-do, South Korea), and the severity of AD was evaluated with the SCORAD index, Eczema Area and Severity Index (EASI), Itch Numeric Rating Scale (NRS), and Dermatology Life Quality Index (DLQI).

**Figure 1:**
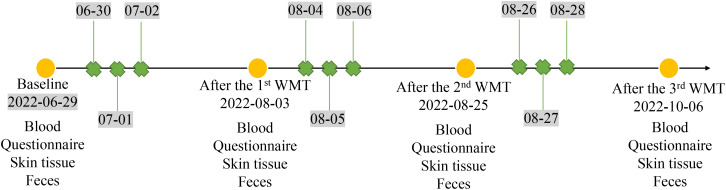
The scheme showing the time points of the WMT procedure and sample collection.

Blood and fecal samples were collected from the patient. In addition, skin swab samples were also collected from both the patient’s skin lesions and his normal skin in the inner elbow region before and after three courses of WMT treatment to conduct a comprehensive analysis of the changes in microbial composition after WMT. The serum eosinophil ratio (EOSR), interleukin-2 (IL-2), IL-4, IL-6, IL-10, interferon (IFN)-γ and tumor necrotic factor-α (TNF-α) levels were determined, and fecal and skin microbial compositions were performed by 16S rRNA sequencing. Community bar plot analysis was used to analyze microbial composition differences. The timeline of WMT procedure and sample collection is shown in **Figure 1**.

**Figure 2:**
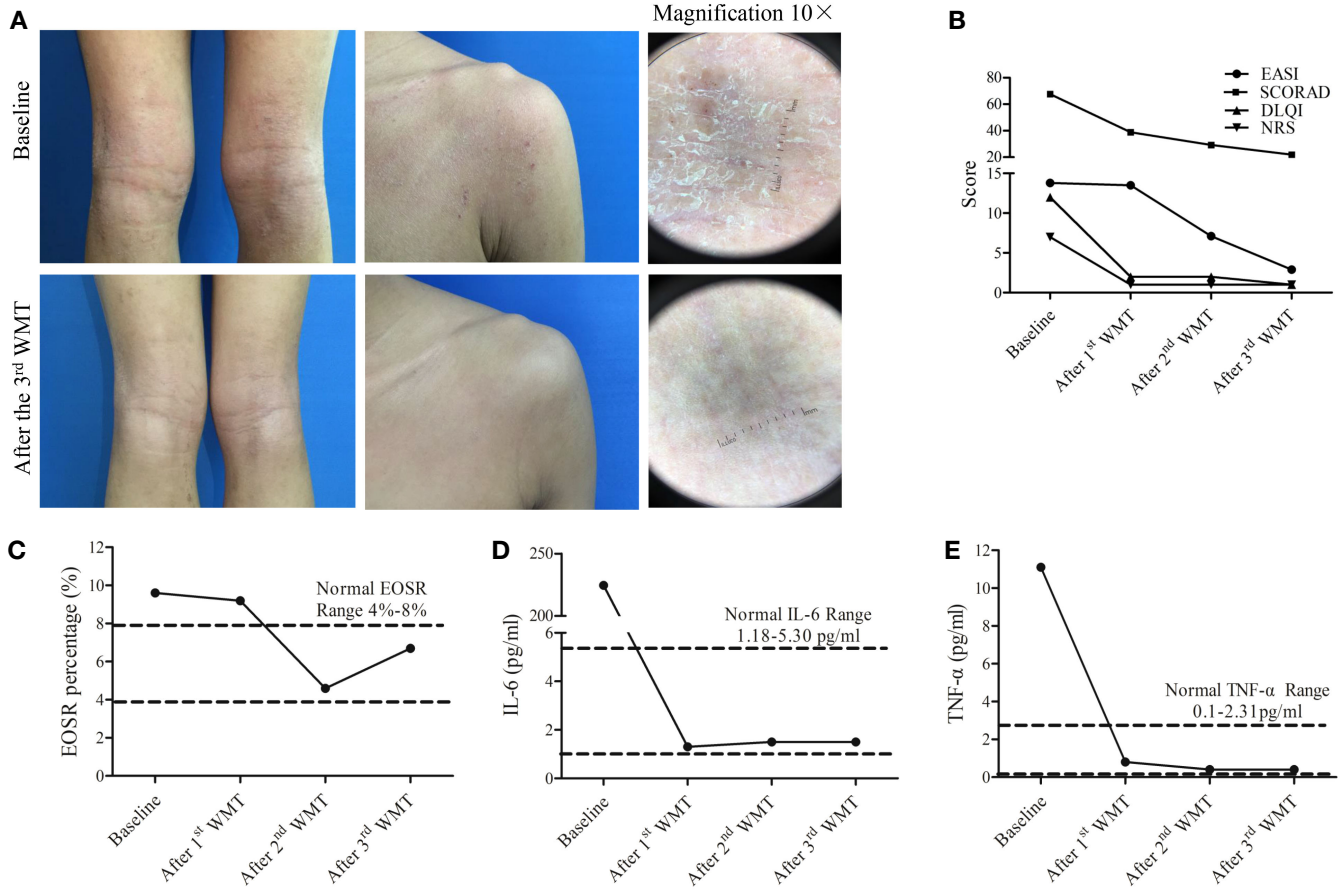
Therapeutic effects of washed microbiota transplantation (WMT) in a 15-year-old patient with atopic dermatitis. **(A)** Clinical images of the patient before and after three courses of WMT. Magnification 10×. **(B)** Scores of SCORAD, EASI, NRS, and DLQI after 1^st^, 2^nd^, and 3^rd^ WMT courses. **(C–E)**. The percentage changes in serum EOSR **(C)**, IL-6 levels **(D)**, and TNF-α **(E)** levels after 1^st^, 2^nd^, and 3^rd^ WMT courses. SCORAD, Scoring Atopic Dermatitis; EASI, Eczema Area and Severity Index; NRS, Numeric Rating Scale; DLQI, Dermatology Life Quality Index; EOSR, eosinophil ratio; IL-6, interleukin-6; TNF-α, tumor necrotic factor-α.

One month after the completion of the three-course WMT treatment, the patient experienced effective control of itching, along with a reduction in the number of skin lesions and improved bowel movements. Moreover, significant improvement was also observed in the patient’s skin after three courses of WMT, with a noticeable decrease in skin rashes and a smoother overall appearance of the skin (**Figure 2A**). The SCORAD, EASI, NRS, and DLQI scores were significantly lower after each WMT course compared to the baseline (**Figure 2B**). Importantly, no adverse events were observed in the three courses of WMT. The patient was not allergic to sesame seeds, animal hair, and pollen after three courses of WMT.

**Figure 3:**
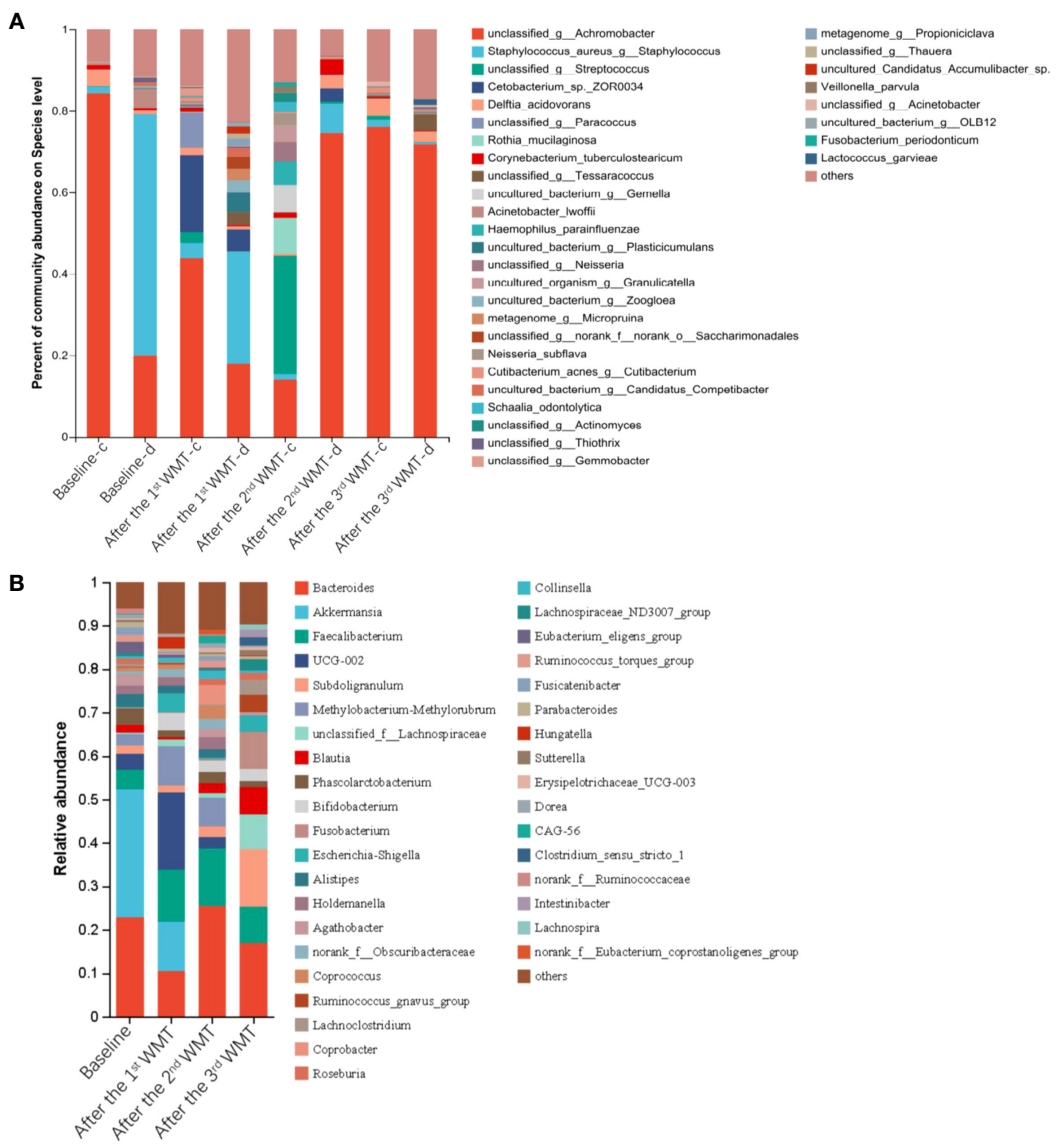
Microbial profiles of the skin and gut in a 15-year-old patient with atopic dermatitis before and after washed microbiota transplantation. **(A)** The abundance of skin microbiota in healthy skin (-c) and lesioned skin (-d). **(B)** The abundance of the gut microbiota.

The serum levels of IL-2, IL-4, IL-10 and IFN-γ were within normal levels before and after WMT (**Supplementary Figure 1**). The serum levels of EOSR, TNF-α, and IL-6 were reduced to normal levels after the third course of WMT (**Figures 2C–E**). Furthermore, community bar plot analysis revealed a significant decrease in the *S. aureus* count in the skin lesions (**Figure 3A**). The intestinal flora was gradually diversified and altered, and the abundance of *Bifidobacterium* species increased significantly after WMT (**Figure 3B**).

The patient was followed up by telephone or hospital visit every month and subjected to evaluation for SCORAD, NRS, and DLQI at each follow-up visit. The above-mentioned indicators were all normal, and the patient was in a stable condition with complete remission at the last follow-up visit on June 1, 2023.

## Discussion

In the present case of a 15-year-old adolescent boy, three courses of WMT treatment resolved clinical symptoms and signs with apparent improvement of serum EOSR, TNF-α, and IL-6 and skin and gut microbiota. Since no topical treatments were used, the positive clinical outcomes observed can be attributed solely to the WMT treatment. To the best of our knowledge, this is the first case report to describe the use of WMT, instead of FMT, as an effective therapy for AD, leading to positive clinical outcomes without obvious adverse events in an adolescent boy.

The role of the gut microbiota in AD has been widely investigated (13). A systematic review demonstrated that while nearly half of included studies reported a positive effect of an altered gut microbial colonization due to the use of probiotics on the severity of AD, other studies did not observe such effect (13). Recently, animal studies have found that FMT treatment benefits mice or dogs with AD by enhanccing gut microbiota homeostasis (7, 14–16). In 2022, Mashiah et al. reported that four courses of FMT treatment achieved a 50% reduction in the SCORAD score in seven (77.8%) of nine adult patients (5 males and 4 females, 24–68 years old) with moderate-to-severe AD (9, 17). After the treatment, two patients had an exacerbation in the SCORAD score, which was alleviated after an additional fifth FMT course, and other two patients with a relapse were treated with a different medication (9). WMT is an improved FMT based on a smart fecal bacterial separation system and strict quality control-related washing processes. Compared to traditional hand prepared FMT, WMT removes pro-inflammatory metabolites with centrifugal washing and thus is relatively safer than FMT (18). In addition to its safety profile, WMT exhibits its clinical efficacy in the treatment of AD in children, as demonstrated in the present case; however, the efficacy needs to be confirmed in well-designed randomized controlled trials.

The pathogenesis of AD involves complex factors, including immune modulation and the gut/skin microbiota (7). AD is characterized by high levels of inflammation, which impacts the diversity of intestinal/skin microbiota, and intestinal/skin microbiota dysbiosis, which, in turn, further exacerbates the inflammatory response (19, 20). Similarly, the impaired skin barrier in AD allows for the colonization of *S. aureus*, which, in turn, stimulates the keratinocytes to produce endogenous proteases, worsening barrier dysfunction (21, 22). *S. aureus* expresses a variety of pathogenic factors that contribute to AD-related inflammation (23–25). It has been reported that the specific binding of staphylococcal protein A of *S. aureus* with TNF receptor 1 induces the release of pro-inflammatory cytokines such as IL-8, thus activating nuclear factor kappa B pathway and stimulating human keratinocytes (26). Animal model experiments have confirmed that FMT can restore gut microbiota and immunologic balance (Th1/Th2) and suppress AD-induced inflammation (7). In the present case, we observed that after each WMT course, the patient’s intestinal flora gradually diversified, with increased bacterial counts of *Bifidobacterium* species. Additionally, the eosinophil ratio and serum levels of pro-inflammatory factors such as TNF-α and IL-6 decreased, along with a decreasing number of *S. aureus* in the skin lesions. These findings suggest a possible relationship between intestinal and skin microflora. By targeting the intestinal-skin axis and inducing alterations in the intestinal microflora, WMT may help reduce inflammation and improve the skin microbiota in patients with AD. This case report highlights the potential effectiveness of WMT as a promising treatment strategy for AD.

It must be acknowledged that this is a single case study, and findings are preliminary and observational and thus, cannot serve as definitive guidelines for clinical management of AD. The implementation of WMT in the treatment of other patients with AD should be carefully considered, taking into account factors such as the AD stage, delivery method and volume, WMT frequency, and other individual concerns. Therefore, it is crucial to continuously update and optimize the treatment protocol of FMT/WMT and regularly monitor and evaluate the long-term treatment efficacy and potential adverse events (27). Moreover, multicenter randomized, double-blind clinical trials and longer-term AD reversal studies are required to formulate a treatment recommendation (28, 29).

In conclusion, WMT treatment successfully resolved clinical manifestation, with the improvement of serum EOSR, TNF-α, and IL-6 and skin and gut microbiota, in an adolescent boy. This case report provides clinical evidence supporting the use of WMT in the treatment of AD, which may be attributed to the regulation of the skin microflora and inflammatory response through the intestinal-skin axis. These findings contribute to the growing body of evidence on the effectiveness of FMT/WMT in AD treatment.

## Data availability statement

The datasets presented in this study can be found in online repositories. The names of the repository/repositories and accession number(s) can be found below: PRJNA1015557 (SRA).

## Ethics statement

This study was approved by the Ethics Committee of The First Affiliated Hospital of Guangdong Pharmaceutical University. The study was conducted in accordance with the local legislation and institutional requirements. Written informed consent for participation in this study was provided by the participants’ legal guardians/next of kin. Written informed consent was obtained from the individual(s), and minor(s)’ legal guardian/next of kin, for the publication of any potentially identifiable images or data included in this article.

## Author contributions

WD: Data curation, Investigation, Writing – review & editing. WC: Writing – original draft. HZ: Validation, Visualization, Writing – review & editing. LW: Validation, Visualization, Writing – review & editing. XH: Project administration, Supervision, Writing – review & editing.

## Supplementary material

Supplementary Figure 1 The dynamic changes in serum IL-2 **(A)**, IL-4 **(B)**, IL-10 **(C)** and IFN-γ **(D)** levels after 1^st^, 2^nd^, and 3^rd^ WMT courses.
