# Supplementary figures and images for: Washed microbiota transplantation: a case report of clinical success with skin and gut microbiota improvement in an adolescent boy with atopic dermatitis
Source: Front Immunol. 2023 Nov 15;14:1275427. doi: 10.3389/fimmu.2023.1275427 (PMC10684772; doi:10.3389/fimmu.2023.1275427)

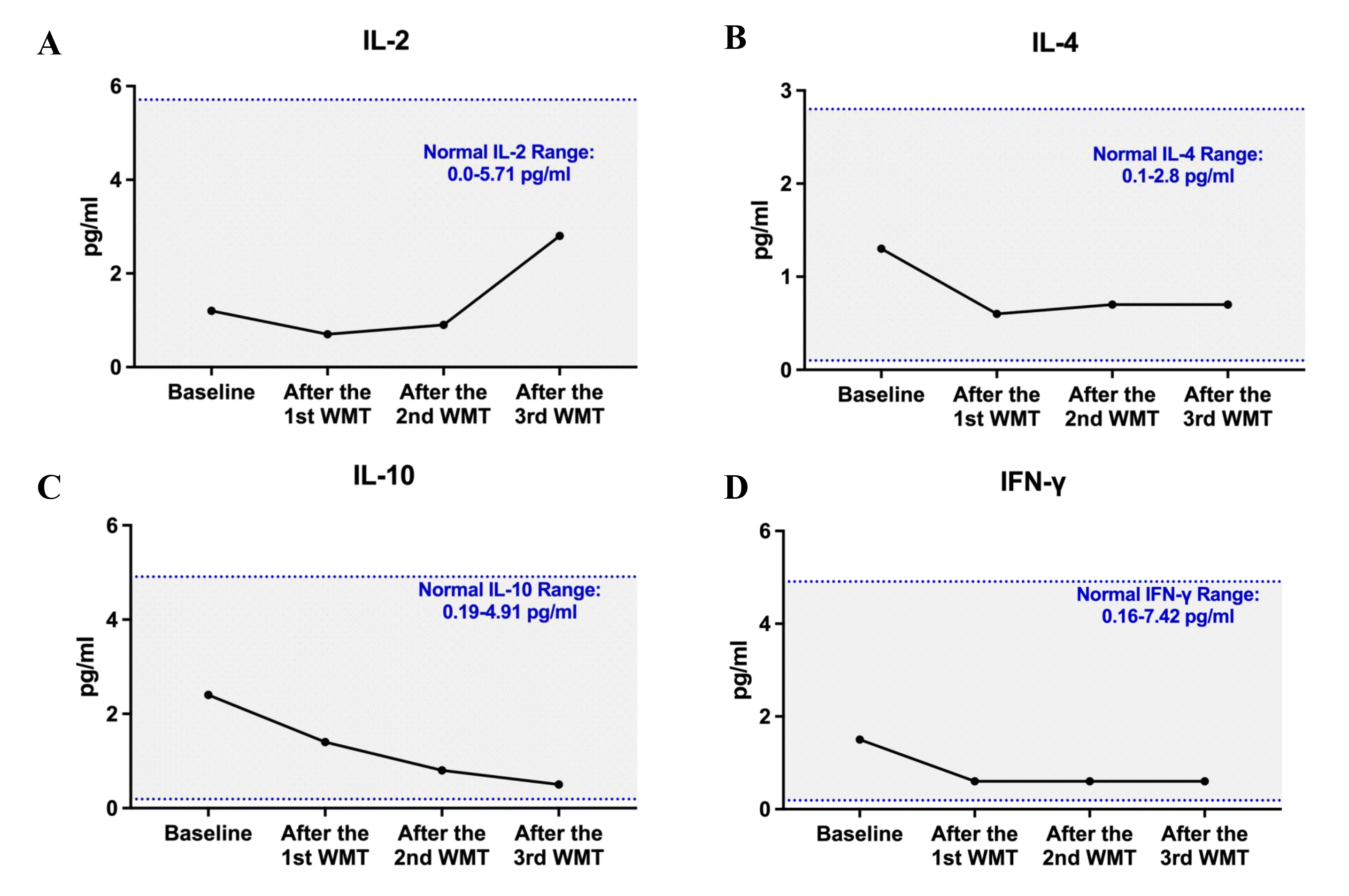

Supplement: Supplementary Figure 1 — The dynamic changes in serum IL-2 (A), IL-4 (B), IL-10 (C) and IFN-γ (D) levels after 1st, 2nd, and 3rd WMT courses. [file Image_1.tif]
